# Establishing the effect of COVID-19 lockdown policy on the utilization of facility-based delivery in Kenya: a multi-method study

**DOI:** 10.1186/s12913-025-13070-4

**Published:** 2025-08-01

**Authors:** MaryBennah N. Kuloba, Christoph Strupat, Thit Thit Aye, Phidelis N. Wamalwa, Judy Gichuki, Benjamin Tsofa, Manuela De Allegri

**Affiliations:** 1 Heidelberg Institute of Global Health, Medical Faculty and University Hospital, Heidelberg University, Heidelberg, Im Neuenheimer Feld 130.3, 69120 Germany; 2 German Institute of Development and Sustainability (IDOS), Tulpenfeld 6, Bonn, 53113 Germany; 3 Department of Health Services, Nairobi County Government, P.O. Box, Nairobi, 45844-00100 Kenya; 4 Health Policy and Systems Research, Kenya Medical Research Institute (KEMRI) -Wellcome Trust Research Programme, Hospital Road, P.O. Box 230, Kilifi, Kenya

**Keywords:** Facility-based delivery, Lockdown policy, Interrupted time series analysis, Pre-post-study, Difference-in-Difference, Pandemic preparedness, Health systems, Facility-based delivery

## Abstract

**Background:**

Amidst the COVID-19 pandemic, lockdown policies emerged as pivotal policies to contain viral transmission. Questions arise about whether their implementation challenged access to care, particularly in regions with fragile health systems, such as sub-Saharan Africa. Robust evidence on the effect of lockdown policies on access to healthcare services is sparse, also due to a lack of suitable data. We addressed this knowledge gap and assessed the effect of the COVID-19 lockdown policy on facility-based delivery during the first wave of the pandemic in Kenya.

**Methods:**

We triangulated results from two parallel yet independent quantitative analyses, exploiting the fact that COVID-19 lockdown policies in Kenya were implemented only in some counties. First, we relied on nationally representative repeated cross-sectional population-based surveys conducted in 2018 and 2020, with data being analyzed using a pre-post study with control. Second, we used monthly data from the Kenya Health Information System (from January 2019 to November 2020) to construct an interrupted time series (ITS) with independent controls, setting April 2020 as the interruption month (i.e., the onset of the lockdowns).

**Results:**

The controlled pre-post-analysis detected no significant effect of the lockdown policy on facility-based delivery in lockdown counties compared with non-lockdown counties. The ITSA model showed that the lockdown counties experienced an immediate increase of 4.97% (CI: 0.51%, 9.43%) in facility-based delivery compared with the non-lockdown counties during the first wave of the pandemic. This was followed by a significant monthly decrease of 0.97% (CI: -1.60%, -0.34%) compared with non-lockdown counties.

**Conclusion:**

We found no overall effect of the lockdown policy on facility-based deliveries. Our findings suggest that when managed effectively, lockdowns do not disrupt access to maternal health services. Our findings highlight the importance of implementing context-specific strategies to safeguard maternal healthcare during public health crises. Future research should explore localized and socioeconomic differences in how populations respond to public health interventions during pandemics.

**Supplementary Information:**

The online version contains supplementary material available at 10.1186/s12913-025-13070-4.

## Background

The COVID-19 pandemic exposed the fragility of healthcare systems in low-income countries and revealed that lockdown strategies predominantly used by high-income countries did not have the same effect as in low-income countries [1, 2]. A modeling study projected at least an 8.4% increase in maternal and a 9.8% increase in newborn mortality per month in low and middle-income countries due to potential disruptions of health systems caused by the pandemic [3]. Countries across the world adopted lockdown policies as temporary mitigation measures to minimize the spread of the COVID-19 virus [4, 5]. Haider et al. defined ‘*Lockdown’* as a set of policies and interventions systematically applied to a broader segment of the population, with equitable enforcement, which included restrictions on social and economic activities aimed at limiting transmission of the virus [5]. These policies excluded mandatory measures aimed at individuals or specific locations, as well as population-wide measures such as wearing facemasks or physical distancing that do not significantly restrict personal freedom or disrupt everyday life [5, 6].

The imposition of lockdown policies carried with them the risk of reversing progress made toward maternal and child health [7]. For instance, global health efforts championed increased facility-based delivery as a gateway to achieving Sustainable Development Goal 3.1, which calls for the reduction of maternal mortality to less than 70 per 100,000 live births [2, 3]. Fear emerged that such lockdown policies, coupled with existing societal, geographic, and economic disparities, could further disable access to health services, particularly for pregnant women, while seeking maternal health services [1, 8].

Studies on the effect of lockdown policies on facility-based delivery have focused primarily on high-income countries [9–11]. Although a few studies have been conducted in low and middle-income countries, study findings reported a decreased utilization of facility-based deliveries leading to adverse maternal outcomes [12–16]. Several studies explain the effect of COVID-19 on facility-based delivery in Kenya [17–20], but very few studies have documented specifically the effect of lockdown policy on facility-based delivery. It is essential to distinguish between COVID-19, which caused fear, service disruptions, and health system strain, and lockdown policies that may have compounded these effects by movement restrictions, imposing curfews, and limiting access to health facilities. We found only three qualitative studies and one quantitative study that tried to assess the effects of the lockdown policy, yielding mixed results [21–24]. A qualitative study by Mattah et al. explored how lockdown policy exacerbated delays in seeking maternal health services in remote marginalized communities, resulting in poor maternal health outcomes [21]. In contrast, a quantitative study by Wambua et al. did not find any effect of the lockdown policy on facility-based deliveries among selected facilities in Kenya [24]. However, these studies are conducted in a restricted geographical area, and therefore, results may not be generalizable.

Our research aims to fill this existing knowledge gap by adopting a multi-method approach to evaluate whether lockdown policies led to a reduction of facility-based deliveries in Kenya. We triangulate results from a pre- and post-analysis with independent controls with results from an interrupted time series with independent controls to generate robust evidence on the effect of lockdown policies on the utilization of maternal healthcare services in Kenya during 2020.

### Study methodology

#### Study setting

Kenya is a lower-middle-income nation and has a population of over 47 million people [25]. Approximately a third of the population lives in poverty, access to health insurance coverage remains low (20%), and out-of-pocket expenditure is estimated at 23% of the total health expenditure [26]. Further, the pandemic pushed an additional 4% of the population below the poverty line, negatively impacting people’s livelihoods [27, 28]. In 2013, the country implemented a devolved system of governance with the national government and 47 semi-autonomous county governments [29].

Kenya’s health system is centered around four tiers of hierarchy: community health, primary health care hospitals, secondary hospitals, and national referral hospitals [30]. Primary healthcare services have been identified as the backbone of a healthy society, especially in developing countries, as it is the point of contact between the health system and communities [30]. Kenya developed the Primary Healthcare Strategic Framework as a pathway to spell out the implementation of the Universal Health Agenda [30]. Free maternal healthcare services were introduced in public hospitals in 2013 to reduce financial catastrophe and increase facility-based delivery [31, 32]. This resulted in an improvement in coverage of maternal indicators, including facility-based delivery (88%), skilled birth delivery (89%), and antenatal care visits (98%) [33]. Despite progress made, maternal mortality is still high at 530 deaths per 100,000 live births [34]. The implementation of lockdown policies during the pandemic disrupted primary healthcare services, despite efforts to sustain the delivery of maternal health services [35].

#### Intervention: implementation of the lockdown policy

The WHO^1^ declared the COVID-19 outbreak a pandemic on 11th March 2020 and Kenya reported its first case on 13th March 2020 [17]. In response to the high morbidity and mortality emerging from the COVID-19 virus, the Kenyan government implemented a COVID-19 lockdown policy that involved geographic lockdowns such as the closure of selected inter-county borders, a ban on international travel, home confinement, including nationwide curfews, assembly restrictions, and closure of businesses [17, 36]. See details on the implementation of the lockdown policy in Kenya in Appendix 1. This paper focuses on geographic lockdown, hereafter referred to as ‘lockdown policy’.

**Fig. 1:**
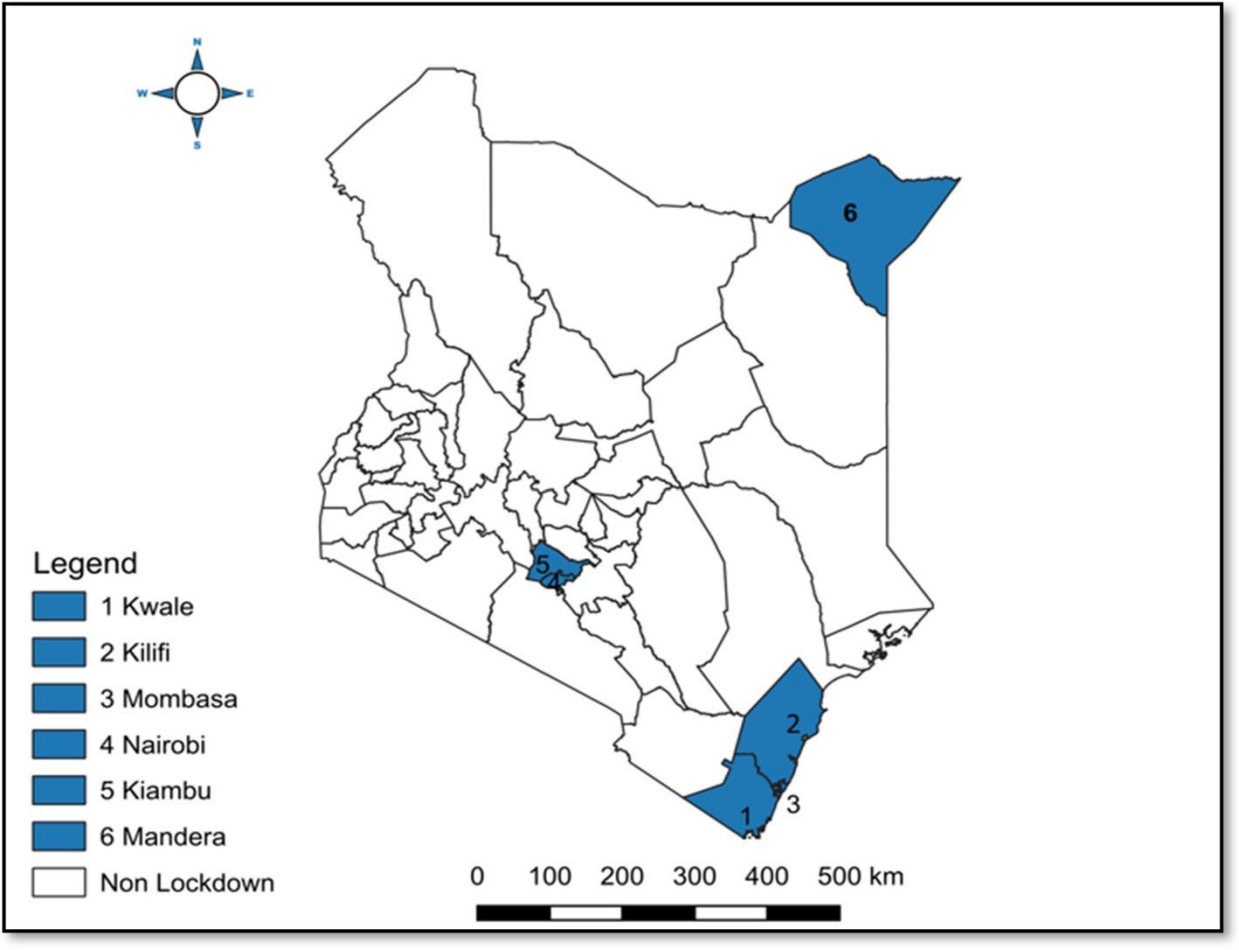
Lockdown and non-lockdown counties

Six counties were purposively placed under a ‘ *Lockdown’* in the period between April to July 2020 [17, 36]. Specifically, Nairobi, Mombasa, Kiambu, Kwale, Kilifi, and Mandera counties, see details Fig. 1, where people were geographically restrained from traveling outside their counties and thus categorized as ‘lockdown counties’ [17, 36]. In contrast, the remaining 41 counties were less restrained and thus considered as ‘non-lockdown counties [5, 17]. The lockdown policies were lifted as of July 2020 [17, 36].

Although pregnant women were permitted to access maternal care during emergencies, they were confronted with structural and social barriers, including limited transportation, fear of infection, economic loss, and movement restrictions [22, 37]. The lockdown policy was implemented in conjunction with other parallel supportive measures to improve maternal care, including the adoption of WHO and Kenya COVID-19 RMNH guidelines, the use of technologies, and other innovative strategies [38, 39].

#### Overall study design

Our study employed a pre-post design with independent controls, analytically referred to as the difference in differences (DID) model, and a controlled interrupted time series analysis (ITSA). The DID model allowed us to isolate the lockdown policy effect by comparing the pre- and post-policy changes between the intervention (lockdown counties) and control group (non-lockdown counties) while accounting for time-invariant unobserved heterogeneity. The controlled ITSA enabled us to identify and quantify immediate changes in the effects of the lockdown policy, as well as month-to-month variations. This approach accounted for temporal trends and correlations within the data. Employing both quantitative analyses in parallel allowed us to leverage their complementary strengths. While DID focuses on differential changes between groups, ITSA offers insights into the timing and progression of those changes. Together, these methods provided a more robust and comprehensive assessment of the lockdown policy’s effect, enhancing the causal inference and ensuring a more nuanced analysis.

### Pre-post study with control: Difference in difference (DID)

#### Study design

A pre-post study with independent controls was employed in the first part of the analysis to assess the effect of the lockdown policy on facility-based delivery. Our study utilized two data points for lockdown and non-lockdown counties, accounting for the pre-policy and the post-policy period.

#### Data sources and samples

Our research utilized population-based household survey data from randomly selected households in Kenya that operate in the informal economy to compare changes in facility-based deliveries before and after the implementation of the lockdown policy [40]. While our study focused on the informal sector households, this demographic constitutes a significant portion of Kenya’s workforce (84%) [41] and contributes approximately 32% of the nation’s GDP [42–44], providing a substantial basis for our analysis. The informal sector, defined as all work conducted within unregistered or incorporated enterprises, is characterized by small businesses and trading [43, 44].

#### Data collection procedures

The sampling procedure for the survey has been described in detail elsewhere [40]. In brief, the survey was designed as a multi-stage stratified cluster probability sample to ensure that every household operating in the informal economy had an equal probability of being selected. Primary sampling units (PSUs) were derived from the 2019 national census data from the Kenya National Bureau of Statistics (KNBS). The sampling process involved stratifying the country into regions, followed by counties, and further into districts and villages. The survey was embedded in another study that evaluated social protection and social integration in the informal sector during the pandemic [40]. For this study, we focus on the second data collection exercise that took place in December 2020. Information on the birth history of the mother was recalled retrospectively. We focused specifically on women who delivered in 2018, 2019, and 2020 [40]. Face-to-face interviews were conducted with the household head and one randomly selected member aged 15 or above [40]. Our sample size includes 632 reported deliveries, out of which 403 deliveries took place during the pre-policy period and 229 deliveries during the post-policy period in both lockdown and non-lockdown counties. Friedrich-Ebert-Stiftung Germany (FES), the International Labour Organization (ILO), and the German Institute of Development and Sustainability (IDOS) coordinated to jointly implement the surveys as cross-sections of informal economy households.

#### Data set up and study variables

Our research focused on facility-based delivery before and after the implementation of the lockdown policy. Subsequently, we categorized deliveries that took place in 2018 and 2019 as pre-policy periods or baseline and those in 2020 as post-policy periods or endline.

#### Outcome variable

Our outcome was defined as the proportion of women who delivered at the facility vs women who delivered at home among all women who experienced a delivery in the survey.

#### Exposure variable and covariates

Our exposure variable was lockdown policy, defined in relation to the county of residence. This was operationalized as a binary variable that differentiated deliveries taking place in lockdown counties and non-lockdown counties. The selection of covariates was guided by a comprehensive review of relevant literature, considering their documented likelihood of facility-based delivery [45, 46] and their availability within the data set. These included covariates such as wealth quantiles, education status, place of residence, and women’s ages. See details on the definition of variables in Appendix 2. Women’s age was modeled as a continuous variable to retain statistical power and avoid arbitrary cut-offs that may reduce precision and obscure underlying trends [47, 48]. To assess the robustness of our findings, we also categorized age into groups (15-23, 24-28, 29-32, and 33-49). The results remained consistent across model specifications. We acknowledge that our dataset did not include other key variables such as parity or birth order, which limited our ability to adjust for these important covariates. We note that, being a DID model, all covariates only increase the precision of the estimate, but do not affect the actual direction of the impact assessments [49, 50].

### Analytical approach and statistical modeling

#### Pre-post study with control

We first employed descriptive statistics to describe sample distribution characteristics between lockdown and non-lockdown counties, including facility-based delivery between lockdown and non-lockdown counties before and after the lockdown policy. Thereafter, we deployed a difference-in-difference estimation to evaluate the effect of lockdown policy on facility-based delivery. The model was based on the following specifications:

The equation was expressed as

$$\begin{aligned}{\gamma }_{ict}=&{\beta }_{\theta }+{\beta }_{1}{l{d}^{2}}_{ct}+{\beta }_{2}{Time}_{t}+{\beta }_{3}\left({ld}_{ct}*{Time}_{t}\right)\\&+{\beta }_{4}{X}_{it}+{V}_{c}+{\varepsilon }_{ict}\end{aligned}$$

- ${\gamma }_{ict}$ is the outcome of interest whether an individual *(i)* living in the counties *(c)* experienced delivery at a given time *(t)*
- ${\beta }_{\theta }$ is a constant term.
- ${ld}_{ct}$ is the lockdown dummy variable showcasing whether the counties *(c)* were in lockdown (1) or non-lockdown (0) counties at a given time *(t).*
- Time is represented as a binary variable assigned a value of 0 for the pre-policy years (2018 and 2019) and 1 for the post-policy year (2020).
- The Difference in difference estimate is the interaction of time and lockdown dummy variables, hence ${ld}_{ct}*{Time}_{t}$ with its ${\beta }_{3}$ coefficient.
- ${X}_{it}$ indicates covariates identified for individual and household characteristics, while *ε* is the error term.

Clustering was done at the county level with county-fixed effects denoted by ${V}_{c}$ to control time-invariant county characteristics. This analysis was conducted using Stata/BE version 17. We compared the results of ordinary least squares (OLS) regression with those of multivariate logistic regression to select the most appropriate model (see details in Appendix 4). As a sensitivity analysis, we further categorized facility-based delivery before April 2020 as pre-policy and after April 2020 as post-policy (see details in Appendix 5).

### Interrupted time series analysis with independent control

#### Study design

We employed an ITSA with independent controls to establish the effect lockdown policy. The lockdown was enforced nationwide, designating counties under lockdown as the intervention group, while those not under lockdown as the control group. Our dataset spanned January 2019 to December 2020, comprising 24 monthly data points, with 15 months for the pre-policy and 9 months for the post-policy. In line with the overall study design, we placed our interruption in April 2020 to quantify the effect of the lockdown policy.

#### Outcome variable

Our outcome variable was defined as the proportion of facility-based delivery. It was calculated as the number of facility-based deliveries (numerator) divided by the estimated total number of deliveries (denominator).

#### Data sources and data setup

This analysis utilized data from Kenya’s Health Information System (KHIS) and the Ministry of Health's annual statistical reports.

#### KHIS data management and imputation of missing data

From the KHIS, we extracted monthly service counts of total deliveries from 3,606 public and private primary health facilities. Total deliveries include normal delivery, assisted vaginal delivery, breech delivery, and cesarean delivery. We addressed outliers in the monthly service counts for all facilities by implementing a rolling modified z-score method within each facility, assessing both the 24 months preceding and following each point in time. Outliers were identified based on a rolling modified z-score threshold exceeding 15. Identified outliers were subsequently corrected using a rolling median method specific to each facility, encompassing the 12 months before and after each observation. Additionally, we addressed internal missing counts for facilities meeting defined criteria: those with no more than 10 consecutive missing values and at least 10 observed data points over the study period. Missing values were imputed within each facility using the local polynomial smoother (LPS), a nonparametric estimator. In this LPS-based regression, a low-degree polynomial is fitted through weighted least squares to local segments of the time series, delimited by a kernel or moving window with a specified bandwidth or smoothing parameter. This imputation approach is suitable for diverse time series trajectories [51, 52] and was selected to align with the heterogeneous patterns observed across institutions. To minimize bias, consistent with established literature on missing data handling [53–57], this approach was applied at the facility level, followed by aggregation at the county level.

#### The Ministry of Health's annual statistical reports

To construct the denominator for facility-based delivery rates, we sourced data on estimated deliveries, total population, and projected population growth rates across 47 counties from the annual statistical reports from 2014 to 2023. Population estimates during the study period were derived from the 2019 census, with annual estimates converted to monthly estimates by dividing by 12. Thereafter, we merged the denominator data and calculated the proportion of facility-based delivery.

#### Variables operationalization

To establish the overall effect of the lockdown policy, we constructed a national-level monthly time series using a two-step process. First, we aggregated monthly service utilization counts from the facilities by county, converting them into percentages using an appropriate denominator for each county. Next, the county-level percentages were aggregated to derive national-level percentages for analysis.

#### Interrupted time series components

Seven interrupted time series components were included in our ITSA model [58]. These are described below in the analytical approach.

#### Analytical approach and statistical modeling

Our ITSA was based on a segmented regression approach to estimate the effect of lockdown policy among primary care facilities in Kenya [58–61]. We first visually plotted the outcome variable over time to examine trends, seasonality, outliers, and functional forms of the variable. Thereafter, we calculated summary statistics of the outcome for the entire study period as well as the sub-period among lockdown and non-lockdown counties during the pre-post-policy period.

Later, we inspected for autocorrelation (ACF) and partial autocorrelation (PACF) plots [58] and conducted the Durbin-Watson test for 12 lags to verify the autoregressive process (AR) and moving average process (MA) [62]. We deployed likelihood-ratio tests to ascertain whether the parameters of the AR and MA processes are sufficient and appropriate [63]. The generalized least square model was used to account for autocorrelation. We utilized a 5% significance level and 95% confidence intervals.

Further, we examined linear and quadratic functional forms to capture the trend in the post-pilot and used the Akaike Information Criteria (AIC) to select the best-fitting model [62].

Our final model included seasonality as a binary variable with months with higher utilization (April to June) coded as 1 and the remaining months as 0 [60]. Our time series model with control adjusted for the AR process at (*p* = *5)* and MA process at *(q* = *1)* was expressed as:

$$\begin{aligned}deliver{y}_{pro}=&{\beta }_{\theta }+{\beta }_{1}time+{\beta }_{2}ld+{\beta }_{3}ld\_time\\&+{\beta }_{4}ld\_level+{\beta }_{5}ld\_trend\\&+{\beta }_{6}level +{\beta }_{7}trend\\&+Seasonality+\varepsilon\end{aligned}$$

- delivery_pro is the outcome variable and represents the proportion of facility deliveries in a specific month.
- ${\beta }_{\theta }$ and ${\beta }_{3}$ represent the period before the policy. Specifically.
- ${\beta }_{\theta }$ and ${\beta }_{1}$ indicate the baseline level (intercept) and pre-existing trend for the non-lockdown counties for the period before the lockdown policy was implemented; $time$ was coded chronologically from 1 to 24 representing months from January 2019 to December 2020 to capture a monthly change for the pre-policy period.
- ${\beta }_{2}$ and ${\beta }_{3}$ indicate differences in level and trend between lockdown and non-lockdown counties before the policy; $ld$ differentiates non-lockdown counties (0) and lockdown counties (1); $l{d}^{3}\_time$ represents the interaction between lockdown counties and time variable.
- ${\beta }_{4}$ to ${\beta }_{7}$ represent the post-policy period
- ${\beta }_{6}$ and ${\beta }_{7}$ estimate changes in the level and trend of the non-lockdown counties for the post-policy period compared with the pre-policy period.
- $level$ is a dummy variable that differentiates the pre-policy period (0) from the post-policy period (1), and it represents immediate changes after the policy for the non-lockdown counties.
- $trend$ is coded as 0 for the pre-policy period and as 1 to 9 for the post-policy period and represents the monthly effect for the non-lockdown counties after the policy.
- ${\beta }_{4}$ and ${\beta }_{5}$ indicate immediate level change and monthly change between the lockdown and non-lockdown counties for the post-policy period.
- $ld\_level$ is the interaction of lockdown counties with the $level$ and represents the immediate effect due to the lockdown policy.
- $ld\_trend$ is the interaction term of lockdown counties with the $trend$ and indicates the monthly effect of the lockdown policy following the introduction of the lockdown policy.
- $Seasonality$ is a dummy variable that distinguishes the months with higher service use (April, May, and June as 1) from others (0), and finally $\varepsilon$ is the error term.

To validate the robustness of our results, we conducted sensitivity tests that included the model without adjusting for health workers strikers and seasonality (see details in Appendix 7); since the lockdown policy was extended to an additional three counties, we classified 9 counties as lockdown counties and the remaining 38 counties as non-lockdown counties (see details Appendix 8). We adjusted for health workers'strikes as a dummy variable in December 2020 (see details Appendix 9), and expressed the model using quadratic terms (see details Appendix 10). Across all our analysis, we maintained our interruption in April 2020. We utilized Stata/BE version 17 for data management and RStudio version 3.3.3. for our ITSA analysis. We conducted our statistical test with a significance level set at 0.05.

### Ethical considerations

Under the guidelines of the Ethics Committee of the Medical Faculty of Heidelberg University, ethical approval for the study was not required since the authors conducting the analysis were not engaged in data collection and had access exclusively to fully anonymized data. A local data collection agency collected data for the population-based survey within the framework of an agreement with IDOS and with permission from NACOSTI (license number NACOSTI/P/19/1408). Data from the KHIS were collected within the framework of routine service delivery, and permission was granted by the Ministry of Health to use the data for analysis.

## Results

We first report results from the pre-post study design with controls, followed by results from the Interrupted Time Series Analysis with Independent Controls.

### Results 1: Pre and post-test design with control

Table 1 summarizes the sample characteristics. The average age of women of reproductive age was around 27 years. Most women were from the poorest households in both 2018/2019 (35.24%) as well as in 2020 (40.61%). Nearly half of the respondents were from households where the household head had at least a primary level of education in both 2018/2019 (53.10%) and 2020 (51.53%). The majority resided in rural areas (68.98% in 2018/2019 and 75.98% in 2020). Insurance coverage was low in both periods, 2018/2019 (21.14%) and 2020 (26.43%).

**Table 1:** Sample descriptive statistics

	Women of reproductive age (15–49 years)	
Year	**2018–2019**	**2020**
No. of observation	403	229
Lockdown status		
Non-lockdown counties	286 (70.97%)	158 (69.0%)
Lockdown counties	117 (29.03%)	71 (31.0%)
Women’s age in years		
Mean	28	27
SD	0.3	0.4
Wealth quantiles		
Poorest	142 (35.24%)	93 (40.61%)
Poorer	86 (21.34%)	50 (21.83%)
Wealthier	94 (23.33%)	48 (20.96%)
Wealthiest	81 (20.10%)	38 (16.59%)
Education status of HH		
No education	38 (9.43%)	28 (12.23%)
Primary	214 (53.10%)	188 (51.53%)
Secondary + above	151 (37.47%)	83 (36.24%)
Place of residence		
Urban	125 (31.02%)	55 (24.02%)
Rural	278 (68.98%)	174 (75.98%)
Insurance status		
No	317 (78.86%)	167 (73.57%)
Yes	85 (21.14%)	60 (26.43%)

Table 2 summarizes the characteristics and distribution of the study sample. We detected no statistically significant difference among respondents across lockdown and non-lockdown counties in both the pre-policy and post-policy periods. In the pre-policy period, comparisons between lockdown and non-lockdown counties showed significant differences in wealth quantiles (*p* < 0.05), place of residence (*p* < 0.001), and insurance status (*p* < 0.011). In the post-policy period, there was a marginally significant difference in wealth quantiles (*p* = 0.054), and a significant difference in household head education status (*p* 0.025), and place of residence (*p* < 0.001).

**Table 2:** Sample characteristics statistics

Year	Pre-policy (2018–2019)			Post-policy (2020)		
	Non-lockdown	Lockdown	*P*-Value	Non-lockdown	Lockdown	*P*-value
No. obs	286	117		158	71	
Home delivery	42 (14.69%)	23 (19.66%)		24 (15.19%)	13 (18.31%)	
Facility delivery	244 (85.31%)	94 (80.34%)	0.218	134 (84.81%)	58 (81.69%)	0.553
Wealth quantiles						
Poorest	89 (31.12%)	53 (45.30%)		56 (35.44%)	37 (52.11%)	
Poorer	59 (20.63%)	27 (23.08%)	0.007*	34 (21.52%)	16 (22.54%)	0.054†
Wealthier	70 (24.48%)	24 (20.51%)		37 (23.42%)	11 (15.49%)	
Wealthiest	68 (23.78%)	13 (11.11%)		31 (19.62%)	7 (9.86%)	
Education status of HH						
No education	23 (8.04%)	15 (12.82%)		14 (8.86%)	14 (19.72%)	
Primary	157 (54.90%)	57 (48.72%)	0.266	80 (50.63%)	38 (53.52%)	0.025*
Secondary + above	106 (37.06%)	45 (38.46%)		64 (40.51%)	19 (26.76%)	
Place of residence						
Urban	58 (20.28%)	67 (57.26%)		23 (14.56%)	32 (45.07%)	
Rural	228 (79.72%)	50 (42.74%)	** < **0.001**	135 (85.44%)	39 (54.93%)	** < **0.001**
Insurance status						
No	235 (82.17%)	82 (70.69%)		115 (73.25%)	52 (74.29%)	
Yes	51 (17.83%)	34 (29.31%)	0.011*	42 (26.75%)	18 (25.71%)	0.870
Women age	**Mean**			**Mean**		
Women age	286 (27.68%)	117 (27.62%)	0.923	158 (27.08)	71 (27.38)	0.741

95% significance levels *** *p* < 0.01, ** *p* < 0.05, * *p* < 0.1

^†^marginal trend toward significance

Table 3 showcases the effect of the lockdown policy on facility-based delivery among women of reproductive age [15–49] years. Both our adjusted and unadjusted findings showed no significant effect of the lockdown policy on facility-based delivery among informal sector workers'households. Detailed results are shown in Appendix 4.

**Table 3:** Effect of lockdown policy on facility-based delivery among women of reproductive age (15–49) years

	Unadjusted			Adjusted		
	**Odds ratio**	***P*– value**	**95% CI**	**Odds ration**	***P*—value**	**95% CI**
Effect of lockdown policy on facility-based delivery	1.136	0.718	(0.569, 2.268)	1.373	0.391	(0.666, 2.830)

### Results 2: Interrupted time series with independent controls

Our analysis included 3,606 primary care health facilities that operated across 47 counties. Of those, 618 facilities operated in six lockdown counties and 2,988 in non-lockdown counties. Table 4 describes the mean proportion of facility-based deliveries before and after the policy implementation in lockdown and non-lockdown counties. Overall, our analysis showed that more facility-based deliveries were reported in lockdown counties (73%) compared with non-lockdown counties (63%). In lockdown counties, facility-based deliveries remained constant (73%) during the pre-policy (Jan 2019 to April 2020) and post-policy period (May 2020 to Dec 2020). Among the non-lockdown counties, facility-based delivery increased from 61% pre-policy to 66% post-policy.

**Table 4:** Monthly mean percentage of facility-based delivery for lockdown and non-lockdown counties in the pre- and post-policy implementation period

		**Monthly mean percentage of facility-based deliveries (%)**		
Counties	Number of facilities	Overall study period	Jan 2019 to April 2020	May 2020 to Dec 2020
Lockdown [6]	618	73.00	73.21	72.75
No-lockdown [41]	2,988	63.00	61.08	66.18

**Fig. 2:**
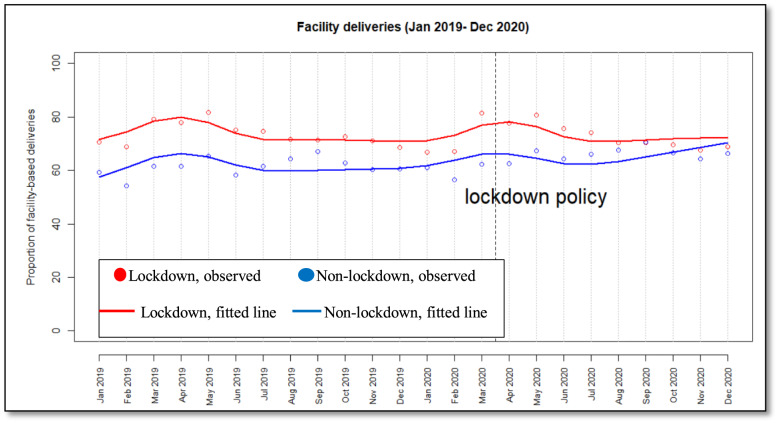
Trends in utilization of facility-based deliveries in primary healthcare facilities

Figure 2 visualizes the utilization of facility-based delivery by plotting both observed (i.e., actual data points) and fitted (i.e., model estimates) lines for lockdown and non-lockdown counties. Pre-policy trends were relatively stable for both lockdown and non-lockdown counties; however, lockdown counties displayed a higher proportion of facility-based deliveries. We do not detect an obvious level change in facility-based delivery in both lockdown and non-lockdown counties immediately following the introduction of lockdown policies. The post-policy trend in lockdown counties was declining until June 2020, thereafter it levelled off. A similar trend was visible among non-lockdown counties; however, it levelled off between June and July 2020, and thereafter it increased steadily to an almost similar level as lockdown counties.

Table 5 shows the results of the selected model after controlling for seasonality. Approximately 72% of facility-based deliveries took place initially in lockdown counties, compared with 58% in non-lockdown counties. The pre-policy trend for facility-based delivery in the non-lockdown counties increased by a monthly rate of 0.3%, while the trend in the lockdown counties decreased by a monthly rate of 0.4% compared with the non-lockdown counties.

**Table 5:** ITSA estimates results for facility-based delivery with seasonality (2019–2020)

Baseline level	**Estimates [95% CI] (100%)**	***P*- value**
Pre-policy period		
Difference between lockdown and non-lockdown counties ($\beta_2$)	14.39 [12.91, 15.87]	*P* < 0.001***
Non-lockdown counties ($\beta_0$)	57.91 [56.77, 59.06]	*P* < 0.001***
Pre-existing trend		
Difference between lockdown and non-lockdown counties$\beta_3$	0.35 [0.53−0.17]	*P* < 0.001***
Non-lockdown counties (${\upbeta }_{1})$	0.26 [0.13, 0.39]	*P* < 0.001***
Post-policy period		
Effect of lockdown policy (Difference between Lockdown and non-lockdown counties)		
Level change ($\beta_4$)	4.97 [0.51, 9.43]	*P* < 0.04*
Trend change ($\beta_5$)	−0.97 [−1.60−0.34]	*P* < 0.001**
Lockdown period (non-lockdown counties)		
Level change ($\beta_6$)	−6.37 [−9.62, −3.12]	*P* < 0.001***
Trend change (${\upbeta }_{7}$)	1.39 [0.94, 1.85]	*P* < 0.001***
Seasonality	7.89 [6.45, 9.32]	*P* < 0.001***

Significance level at, 0.01^*^, and 0.05^**^, 0.001 ^***^respectively

Immediately following the policy implementation, facility-based deliveries in the lockdown counties increased by approximately 5% compared with the non-lockdown counties. However, after the initial increase, the proportion of facility-based deliveries in lockdown counties decreased significantly by 1% each month compared with the non-lockdown counties.

## Discussion

Our study makes a valuable contribution to the literature as the first in Kenya to examine the effect of lockdown policies on facility-based deliveries using a multi-method approach, employing two parallel quasi-experimental techniques: a controlled pre-post analysis and a controlled ITSA. Unlike previous research focused on the broader pandemic impact, we examined specifically the effect of lockdown policies, offering a distinct perspective compared with available evidence [17, 18]. Leveraging the complementary strengths of DID and ITSA allowed us to gain an in-depth understanding of the effect of lockdown policies on access to facility-based delivery. DID provided a broad overview by estimating the average treatment effect and controlling for time-invariant confounders. In contrast, ITSA provided detailed temporal insights and allowed us to control for secular trends. Triangulating findings across the two methods enables us to provide a more nuanced understanding of the policy’s effect on maternal health services, minimizing bias and enhancing the credibility of our policy recommendations. The higher proportion of facility-based deliveries in lockdown counties compared to non-lockdown counties is consistent across descriptive and inferential findings from both the ITSA and DID models. We have no means of explaining it if not attributing it to contextual factors that would need to be explored qualitatively. Since the difference was present before the intervention (i.e., introduction of lockdown polices) and affects the overall level and not the trend, it does not jeopardize any of the modelling assumptions necessary for impact estimation.

We start our discussion by examining the overall effect of lockdown policies on facility-based delivery. Appraising findings across the two types of analyses together, we note that the implementation of the lockdown policy did not yield any effect on the utilization of facility-based delivery. Although findings from the ITSA portray a more complex narrative, suggesting an initial increase in service use followed by a sustained decrease over time, this result was not confirmed across all sensitivity analyses and should be interpreted with caution. Despite this complexity, the cumulative effect aligns with that of the DID model. Ultimately, we detect no effect in the utilisation of facility-based deliveries between lockdown and non-lockdown counties.

Different factors may explain the stability of facility-based deliveries in Kenya despite the imposition of lockdown policies in some counties. First and foremost, pregnant women were exempted from the lockdowns and could continue to move if in need, even after curfew [17, 18]. It is clear from our data that they did so, preferring to deliver in the presence of a health worker rather than at home. Previous literature showed that pregnant women showed commitment to deliver in health facilities because of their fear of contracting COVID-19 in community settings and by having preplanned their deliveries [18, 64, 65]. Drawing on Kenya’s experiences with previous crises (e.g., floods, terrorism, health workers'strike) [66–68]. The government and health authorities leveraged existing strategies. For instance, the midwifery-led care model helped to decongest health facilities and ensured that pregnant women had access to facility-based deliveries despite challenges posed by the lockdown policy [66, 67, 69]. The government also sought collaboration from healthcare workers and community health workers to sustain access to health facilities during COVID-19 times, proving its investments were successful [70, 71]. Evidence from the literature indicated that community health workers who received targeted training aligned with WHO guidelines for the COVID-19 response were able to sustain the coverage and continuity of community-delivered care during the pandemic [72]. Similar findings were also reported in studies conducted in Liberia, Mali, Malawi, and Uganda [73, 74]. Leveraging well-established platforms such as community health models proved to be a critical strategy in protecting vulnerable populations from unexpected shocks in the health system. Contrary to our findings, in Uganda, Musoke et al. reported reduced use of facility-based delivery due to the imposition of lockdown policies, as no alternative strategies were instituted, such as CHWs for home visitation to ensure continuity of care [75].

The Kenyan government deployed an additional 5000 healthcare workers to bolster the national COVID-19 response in May 2020 [76, 77]. This expansion of the healthcare workforce facilitated the restoration of maternal health services, enabling providers to resume their primary roles. Consequently, this intervention likely played a crucial role in mitigating disruptions to maternal health services within the research setting. Our findings align with those of Mhajabin et al. (2022) in Bangladesh, who also reported no significant changes in maternal health services due to the lockdowns, attributing it to the additional health force deployed to support the COVID-19 response [78].

Moreover, one also needs to consider that to sustain high maternal care use, Kenya has operated a free maternal care program since 2013 [79]. By ensuring access to delivery services free of charge at the point of use, this program probably acted as an enabler, allowing women to seek care even during the difficult COVID times when resources for care-seeking might have been constrained due to the economic crisis [80]. Our findings are consistent with Wambua et al., who also reported no effect of the COVID-19 lockdown policies on facility-based deliveries [24, 80]. While they further disaggregated their data by private and public facilities, something which our data does not allow for, they observed a significant number of deliveries in private facilities compared with public facilities, a finding that extends beyond the scope of this research. It is possible that high facility-based delivery in private facilities may have potentially offset any reductions in public facilities and contributed to the overall stability in facility-based delivery [24]. Further research is warranted to evaluate the role of the private sector during emergencies to enhance efficient service delivery.

While the initial public health measures and existing maternal health programs may have sustained facility-based delivery temporarily, the subsequent significant monthly decline suggests the emergence of cumulative barriers that intensified over time.

Government-imposed night curfews and movement restrictions limited pregnant women’s ability to access health facilities, particularly during the nighttime when labor often begins. This led some women to opt for home deliveries or seek assistance from traditional attendants, with a potential to result in adverse health outcomes [21, 81, 82]. The reallocation of healthcare resources to manage COVID-19 cases led to reduced availability of maternal health services [19]. Some facilities were repurposed as isolation centres, and staff shortages due to illness or industrial strikes further compromised service delivery [81]. For instance, in Tana River, a maternity wing was converted into an Isolation Ward, while in Mombasa County, maternity services were suspended when Tudor Hospital was designated as an isolation centre. These changes created confusion among pregnant women, who were uncertain about where to seek services [81].

Moreover, the pandemic-induced economic downturn resulted in reduced household income, making it financially challenging for families to afford transportation or the associated costs of facility-based deliveries. This economic strain was particularly acute in informal settlements, where many residents relied on daily wages [19, 83]. Women reported prioritizing essential provisions such as food over healthcare expenses, thereby reducing access to healthcare. Concerns about contracting COVID-19 within a healthcare setting deterred women from seeking maternal health services [19, 83]. Mandatory mask-wearing and the fear of mandatory quarantine upon testing positive further discouraged facility visits [19, 83]. Fear of victimization for violating lockdown curfews and policy brutality, particularly in urban slums, discouraged pregnant women and their caregivers from seeking facility-based delivery services [21, 23, 82]. A study in Nairobi and Kiambu counties found that half of the respondents indicated a decline in trust in healthcare systems due to the pandemic, which was associated with increased likelihood of care avoidance and feelings of unsafety when accessing care [84]. This pattern aligns with findings from other low and middle-income countries, where initial resilience in healthcare utilization gave way to declines as the pandemic persisted [3, 23].

We did not examine COVID-19 prevalence across lockdown and non-lockdown counties. Our focus was to assess the causal effect of the lockdown on facility-based deliveries. While COVID-19 prevalence may have influenced health-seeking behavior, isolating its independent effect was beyond the scope of our analysis. We recommend that future studies incorporate granular COVID-19 prevalence data to better understand its impact on healthcare utilization during health emergencies.

### Policy implications

Our findings underscore the need for targeted and context-specific interventions that address both immediate and long-term measures during public health emergencies. This study highlights how health systems can be resilient to shocks when purposeful strategies aimed at protecting access are set in place ex-ante. These strategies should be maintained in times of crisis, even in the presence of policies such as lockdowns that pose the potential of acting as an extreme disturbance to routine health system operations. The evidence generated by our study suggests that Kenya has been successful in its attempt to protect mothers and their unborn babies in times of crisis and should continue on this path. Efforts should continue to focus on strengthening existing community-based platforms such as midwifery-based care models and referral systems to enhance access to maternal health services, especially during crises/pandemics.

### Methodological considerations

We employed a multi-method triangulation technique, conducting parallel DID and ITSA to strengthen the validity and robustness of our findings. We leveraged nationally representative datasets, including a population-based household survey of informal sector households, and routine health information system (HIS) data.

While the study offers critical insights into maternal health services utilization among women in informal sector households, caution is warranted due to the relatively small sample size, which may limit the generalizability of the findings. Similarly, extrapolation of the DID results to populations in formal employment sectors or urban settings should be undertaken carefully, as these groups may have different healthcare access dynamics. Nevertheless, focusing on informal sector households allowed us to address important equity considerations, given the greater vulnerabilities faced by this population during crises.

Given the limitations in HIS data quality, we employed local polynomial regression to account for non-linear trends and conducted sensitivity analyses to test the robustness of our results. Not all sensitivity analyses confirmed the main findings, suggesting that the initial significant increase in facility-based deliveries should be interpreted with caution. Differences between facility-based delivery estimates from the ITSA and DID analysis, as well as from population-based data, stemmed from our use of county reports as the denominator rather than facility-level data [33]. Temporal discrepancies between DID (2018–2020) and ITSA (2019–2020) data sources may also affect comparability; however, our findings were confirmed through rigorous sensitivity analyses for both pre-post with control and Interrupted time series (ITSA).

The nationwide scope of our ITSA study prevented us from identifying regional differences. Future research should explore how policy impacts differ across counties with varying needs and subnational contexts. Finally, although this study focused on access to care, we were unable to assess the policy impact on the out-of-pocket expenditure due to data limitations. The potential increase in financial barriers during the pandemic highlights a critical gap and underscores the need for strengthened social health protection policies.

## Conclusion

This paper offers unique insights into maternal healthcare utilization in Kenya during the initial wave of the COVID-19 pandemic, concluding that the implementation of lockdown policies had no significant overall effect on facility-based deliveries. The experience of Kenya offers a lesson that, when managed effectively, lockdown policies do not inherently disrupt maternal health services. Our findings highlight the importance of carefully balancing the introduction of restrictive policies against pre-existing policy action and concrete strategies to ensure that access to care is maintained for essential services targeting vulnerable groups. Due to data availability, our research falls short of examining regional responses as well as distributional effects in detail, leaving ample space for future research in this direction.

## Supplementary Information

Supplementary Material 1.

## Data Availability

The Population household data were made available by the German Institute of Development and Sustainability and can be accessed upon request. Request for access to the DHIS data should be directed to the Ministry of Health, Kenya. Data will be made available upon formal request, subject to approval by the Ministry of Health.

## Abbreviation

Ld: Lockdown policy

## Footnotes

1 WHO = World Health Organization.
