# Correction: Establishing the effect of COVID-19 lockdown policy on the utilization of facility-based delivery in Kenya: a multi-method study

**DOI:** 10.1186/s12913-025-13360-x

**Published:** 2025-08-15

**Authors:** MaryBennah N. Kuloba, Christoph Strupat, Thit Thit Aye, Phidelis N. Wamalwa, Judy Gichuki, Benjamin Tsofa, Manuela De Allegri

**Affiliations:** 1 Heidelberg Institute of Global Health, Medical Faculty and University Hospital, Heidelberg University, HeidelbergIm Neuenheimer Feld 130.3, Heidelberg, 69120 Germany; 2 German Institute of Development and Sustainability (IDOS), Tulpenfeld 6, Bonn, 53113 Germany; 3 Department of Health Services, Nairobi County Government, P.O. Box 45844-00100, Nairobi, Kenya; 4 Health Policy and Systems Research, Kenya Medical Research Institute (KEMRI) -Wellcome Trust Research Programme, Hospital Road, P.O. Box 230, Kilifi, Kenya

**Correction** **:** **BMC Health Services Research 25, 1014 (2025)**

**https://doi.org/10.1186/s12913-025-13070-4**

In this article, the author name Manuela De Allegri was incorrectly written as Manuela De Allergi. The author group has been updated above and the original article has been corrected.

