# Supplementary material for: Establishing the effect of COVID-19 lockdown policy on the utilization of facility-based delivery in Kenya: a multi-method study
Source: BMC Health Serv Res. 2025 Aug 1;25:1014. doi: 10.1186/s12913-025-13070-4 (PMC12315436; doi:10.1186/s12913-025-13070-4)
Supplement: Supplementary file 1 — Supplementary Material 1. [file 12913_2025_13070_MOESM1_ESM.pdf]

Supplementary Materials

Decision Minor

Reviewer comment:
1. We notice you have included Appendices in your manuscript. Please ensure this is uploaded as the 'Supplementary Material' file type for it to be considered. This file should be clearly labelled.

Supplementary materials

Changes made: Appendices section moved to Supplementary materials section.

Author Response:

Appendix: 1 Implementation of Lockdown Policy in Kenya.

Classification	Mitigation strategy	Duration
Geographic lockdown	Ban on all international passenger flights	March 2020
	Cessation of movement into and out of Nairobi, Mombasa, and Mandera (except for movement of food supplies and other cargo)	April 2020 – July 2020
	Cessation of movement into and out of Kilifi and Kwale (except for movement of food supplies and other cargo)	April 2020 – June 2020
	Cessation of movement into and out of Old Town in Mombasa and Eastleigh in Nairobi	May 2020 -July 2020
	Cessation of movement into and out of Nairobi, Kajiado, Machakos, Kiambu, and Nakuru counties (except for movement of food supplies and other cargo)	April 2021
	Cessation of movement of persons and vehicles across the Kenya-Somalia and Tanzania International borders, except for cargo vehicles.	May 2020
	Extension of the ban on international flights for 30 days, except for flights evacuating foreign nationals and cargo	April 2020
	Lifting of cessation of movement into and out of Nairobi, Kajiado, Machakos, Kiambu, and Nakuru counties	May 2021
	Lifting of the intercountry cessation of movement in and out of Nairobi, Mombasa, and Mandera, effective 7 July 2020	6th July 2020
	Resumption of local air travel under strict guidelines and protocols	15th July 2020
	Resumption of international air travel under strict guidelines and protocols	1st August 2020
	Restrictions on passengers using public transport to 60% capacity	March 2020
Assembly restrictions and closure of business	Ban on all social gatherings and conferences of an international nature in Kenya	March 2020
	The informal business remains open, complying with physical distancing measures	March 2020
	Schools, colleges, universities, religious structures, non-essential commerce, and outdoor spaces and amenities closed	April 2020
	Restaurants remain open (takeaways); a ban on opening bars (however, lifted after 30 days)	March 2020
	Regulation on the use of public transport to 60% capacity	March 2020

	Requirement for 1.5 meters of physical distance in a public place	March 2020
	phased re-opening for congregational worship at places of worship, weddings Funerals	July 2020
	Signing out for all bars and eateries joints at 10:00 PM daily	September 2020
	An increase of one-third of normal sitting in places of worship	September 2020
	Participation in funerals and weddings increased from 100 to 200	September 200
	Phase - the reopening of schools, beginning with examination classes, Grade 4, Class 8, and Form 4 students	October 2020
	All bars, restaurants, and other public places open to close by 21:00 hours	November 2020
	Political gatherings were suspended for 60 days. However, meetings can take place at one-third capacity in the town hall	November 2020
	Basic learning resumes in person	January 20221
	Working from home, except for essential workers	March 2020
	Implementation of a nationwide curfew between 1900hrs to 0500hrs	March 2020
	Extension of national curfew for 21 days	April 2020
	Extension of nationwide curfew from 21h00 to 04h00 daily for another 30 days.	July 2020
	Nationwide curfew in force throughout the territory of Kenya for a further 60 days between 21h00 to 23h00;	September 2020
	Nationwide curfew extended to 3 January 2021 and to be enforced between 10h00 and 04h00.	November 2020
**Home confinement**	quarantine and care for infected people or suspected cases and their contacts	March 2020
**COVID-19 Screening**	Mandatory screening at all points of entry	March 2020
	Requirements for screening in all public places and buildings	March 2020
**COVID-19 Testing**	Testing and contact tracing	March 2020
	Truck driver required for COVID-19 testing. Negative results were allowed in the country	May 2020
**Sanitation**	Requirement for soap, water, and hand sanitizers in public areas and cough hygiene	March 2020
	Mandatory wearing of face masks in public areas	April 2020

Appendix 2 Definition of variables

Variables	Definition	Measurement
	**Outcome**	
**Facility-based delivery**	Differentiated if a delivery was home or facility-based deliveries	Home delivery (0)<br>Facility-based (1)
	**Exposure**	
**Lockdown policy**	Differentiated counties into lockdown and non-lockdown counties	Non-lockdown (0)<br>lockdown (1)
	**Time/Year**	
**Time (Birth year)**	Differentiated time into pre-policy (2018/2019) and post-policy (2020)	Pre-policy (0)<br>Post-policy (1)
	**Covariates**	
**Wealth quantiles**	Classification of households by wealth status related to the sample, with the least being the poorest	Poorest (1)<br>Poorer (2)<br>Wealthier (3)<br>Wealthiest (4)
**Education status**	The highest education status of the household head	No school (0)<br>Primary (1)<br>Secondary + above (2)
**Place of residence**	Differentiated households lived in urban or rural areas	Urban (0)<br>Rural (1)
**Insurance status**	If the household level is insured	No (0)<br>Yes (1)
**Woman's age**	Mother’s age	Continuous

Appendix 3 Heterogeneity: Facility-based delivery by Time, Socio-economic, and Lockdown counties: Descriptive Statistics

	Non-Lockdown counties			Lockdown counties		
**Year**	2018-2019 Pre-policy	2020 post-policy	P-Value	2018-2019 Pre-policy	2020 post-policy	P-Value
Facility-based delivery	N=244	N=134		N=94	N=58	
Wealth quantiles						
Poorest	63(25.82%)	41(30.60%)		36(38.30%)	28(48.28%)	
Poorer	53(21.72%)	27(20.15%)	0.686	23(24.47%)	13(22.41%)	0.665
Wealthier	61(25.0%)	35(26.12%)		23(24.47%)	11(18.97%)	
Wealthiest	67(27.46%)	31(23.13%)		12(12.77%)	6(10.34%)	
Education status						
No education	13(5.33%)	8(5.97%)		10(10.64%)	9(15.52%)	
Primary	131(53.69%)	64(47.76%)	0.544	43(45.74%)	31(53.45%)	0.271
Secondary + above	100(40.98%)	62(46.27%)		41(43.62%)	18(31.03%)	
Place of residence						
Urban	55(22.54%)	23(17.16%)		60 (63.83%)	31(53.45%)	
Rural	189(77.46%)	111(82.84%)	0.212	34 (36.17%)	27(46.55%)	0.206
Insurance status						
No	198(81.15%)	94(70.68%)		64(68.82%)	39(68.42%)	
Yes	46(18.85%)	39(29.32%)	0.022	29(31.18%)	18(31.58%)	0.960
	Mean	Mean		Mean	Mean	
Women age	244(27.51)	134(27.02)	0.444	94(27.43)	58(27.15)	0.786

Appendix 4 Main results of DID analysis pre-policy (2018-2019) and post-policy (2020)

**Variables**	**Unadjusted** Logistic 0R (95%CI)	**Adjusted** Logistic 0R (95%CI)	**Adjusted** OLS Coff. (95%CI)
Lockdown (0/1)	0.70 (0.23, 2.17)	0.69(0.23, 2.027)	
Time (years) (0/1)	0.96 (0.58, 1.60)	1.06(0.59, 1.92)	0.02(-0.05, 0.09)
Lockdown # time	1.14(0.57, 2.27)	1.37(0.67, 2.83)	0.04(-0.05, 0.13)
Wealth quantiles (Ref: Poorest)			
Poorer (0/1)		1.55(0.88, 2.71)	0.01(-0.09, 0.10)
Wealthier (0/1)		2.47**(1.21, 5.04)	0.06(-0.02, 0.15)
Wealthiest (0/1)		7.97**(1.55, 41.05)	0.10*(-0.00, 0.20)
Education level (Ref: No school)			
Primary (0/1)		2.02**(1.04, 3.94)	0.05(-0.09, 0.19)
Secondary + above (0/1)		4.55***(1.47, 14.09)	0.11(-0.05, 0.26)
Place of residence (Ref: Urban)			
Rural (0/1)		0.34***(0.16, 0.70)	-0.13**(-0.24, -0.02)
Insurance status (Ref: No)			
Yes (0/1)		1.23(0.65, 2.36)	0.03(-0.04, 0.11)

The woman's age		0.98(0.94, 1.02)	-0.00(-0.01, 0.00)
No. observation	632	629	629

OR= Odds ratio; CI=Confidence interval; Coff=Coefficient.
95% significance levels *** p<0.01, ** p<0.05, * p<0.1

Appendix 5 Sensitivity analysis with DID before April 2020 (pre) vs. from April 2020 (post)

**Variables**	**Unadjusted** Logistic 0R(95%CI)	**Adjusted** Logistic 0R (95%CI)	**Adjusted** OLS Coff. (95%CI)
Lockdown (0/1)	0.67(0.21, 2.14)	0.67(0.24, 1.90)	
Time (month) (0/1)	0.81(0.48, 1.35)	1.01(0.55, 1.83)	0.022(-0.056, 0.10)
Lockdown # time	1.42(0.67, 3.04)	1.66(0.76, 3.63)	0.02(-0.08, 0.12)
Wealth quantiles (Ref: Poorest)			
Poorer (0/1)		1.54(0.88, 2.70)	0.01(-0.08, 0.10)
Wealthier (0/1)		2.46**(1.21, 5.04)	0.06(-0.02, 0.15)
Wealthiest (0/1)		7.87**(1.52, 40.82)	0.10*(-0.00, 0.20)
Education level (Ref: No school)			
Primary (0/1)		2.03**(1.04, 3.95)	0.05(-0.09, 0.19)
Secondary + above (0/1)		4.61**(1.49, 14.27)	0.11(-0.05, 0.26)
Place of residence (Ref: Urban)			
Rural (0/1)		0.34***(0.16, 0.69)	-0.13**(-0.24, -0.02)
Insurance status (Ref: No)			
Yes (0/1)		1.25(0.66, 2.40)	0.036(-0.04, 0.11)
Woman's age		1.0(0.94, 1.02)	-0.00(-0.01, 0.00)
No. observation	632	629	629

OR= Odds ratio; CI=Confidence interval; Coff=Coefficient.
95% significance levels *** p<0.01, ** p<0.05, * p<0.1

Appendix 6 Imputation of missing data using local polynomial regression

No. counties	Overall number of facilities	No. facilities for which the criteria do not hold	No. facilities imputed internal missing using local polynomial regression within each facility
Lockdown (6)	2057	1439	618
Non-lockdown (41)	8657	5669	2988
Total (47)	10714	7108	3606

The second supplementary file to the manuscript "Establishing the effect of COVID-19 lockdown policy on utilization of facility-based delivery: A Multi-method study approach."

This supplementary file provides a summary of the results of the sensitivity analyses stated in our manuscript. In addition, it presents the model estimates to the sensitivity analyses and the corresponding graphs of the model outputs. First, without adjusting for both seasonality and the health worker strike in December 2020. Approximately 75% % of facility-based deliveries took place initially in lockdown counties, compared to 61% in non-lockdown counties. The pre-policy trend for facility-based delivery in the non-lockdown counties increased with a monthly rate of 0.1%, while the trend in the lockdown counties decreased by a monthly rate of 0.3% compared to the non-lockdown counties. Immediately following the policy implementation, facility-based deliveries in the lockdown counties relatively increased by nearly 3% compared to the non-lockdown counties, although it was not significant. However, after the initial increase, the proportion of facility-based deliveries in lockdown counties decreased significantly by 1% each month compared to the non-lockdown counties.
Section 1 presents the model estimates and the corresponding graph of the first sensitivity analysis.

Appendix 7 Model estimates without seasonality and the health worker strike

**Pre-policy**		
**Baseline level**	**Estimates [95% CI] (100%)**	**P- value**
Difference between lockdown and non-lockdown counties (β_2)	14.09 [-12.68, -15.49]	P < 0.00***
Non-lockdown counties (β_0)	60.81 [59.82, 61.80]	P < 0.00***
**Pre-existing trend**		
Difference between lockdown and non-lockdown counties (β_3)	-0.30 [-0.47, -0.13]	P < 0.00**
Non-lockdown counties (β_1)	0.11 [-0.02, 0.23]	P < 0.09
**Post-Policy**		
Effect of Lockdown policy (Difference between lockdown and non-lockdown counties)		
Level change (β_4)	2.96 [-1.03, 6.94]	P < 0.15
Trend change (β_5)	-0.72 [-1.21, -0.24]	P < 0.01**
**Lockdown period (non-lockdown counties)**		
Level change	-0.15 [-2.97, 2.67]	P < 0.92
Trend change (β_7)	0.58 [0.24, 0.92]	P < 0.00**

Significance level at, 0.01*, and 0.05**, 0.001 ***respectively

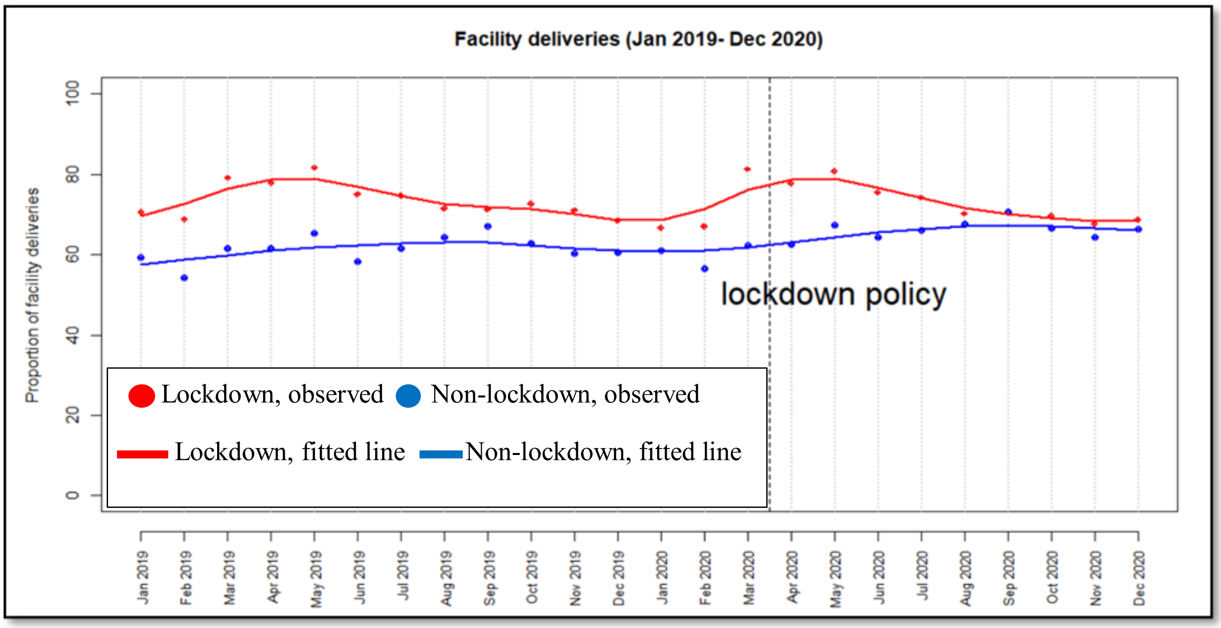

Second, we extended the lockdown policy to three additional counties and thus we classified 9 counties as lockdown counties and the remaining 38 counties as non-lockdown counties. Compared to non-lockdown counties, the size of the immediate effect due to lockdown policy reduced from 5% to 4% and the p-value remained significant. In addition, the monthly reduction in lockdown counties compared to non-lockdown counties remains constant at 1% and is significant, which has been confirmed in our primary model.

Appendix 8 Model estimates the extension of the lockdown policy to Kajiado, Nakuru, and Machakos as intervention counties (January 2019 to December 2020)

**Pre-policy**		
**Baseline level**	**Estimates [95% CI] (100%)**	**P- value**
Before the lockdown policy		
Difference between lockdown and non-lockdown counties (β_2)	10.34[9.29, 11.40]	P < 0.00***
Non-lockdown counties (β_0)	60.83[60.08, 61.58]	P < 0.00***
**Pre-existing trend**		
Difference between lockdown and non-lockdown counties (β_3)	-0.21[-0.34, -0.09]	P < 0.00**
Non-lockdown counties (β_1)	0.09[-0.00, 0.18]	P < 0.06
**Post-policy**		
**Effect of lockdown policy (Difference between lockdown and non-lockdown)**		
Level change (β_4)	3.83[0.81, 6.85]	P < 0.02*
Trend change (β_5)	-0.62[-1.00, -0.24]	P < 0.00**
**Lockdown period (non-lockdown counties)**		
Level change (β_6)	-0.56[-2.69, 1.58]	P < 0.61
Trend change (β_7)	0.68[0.41, 0.95]	P < 0.00***

significance level at, 0.01*, and 0.05**, 0.001 ***respectively

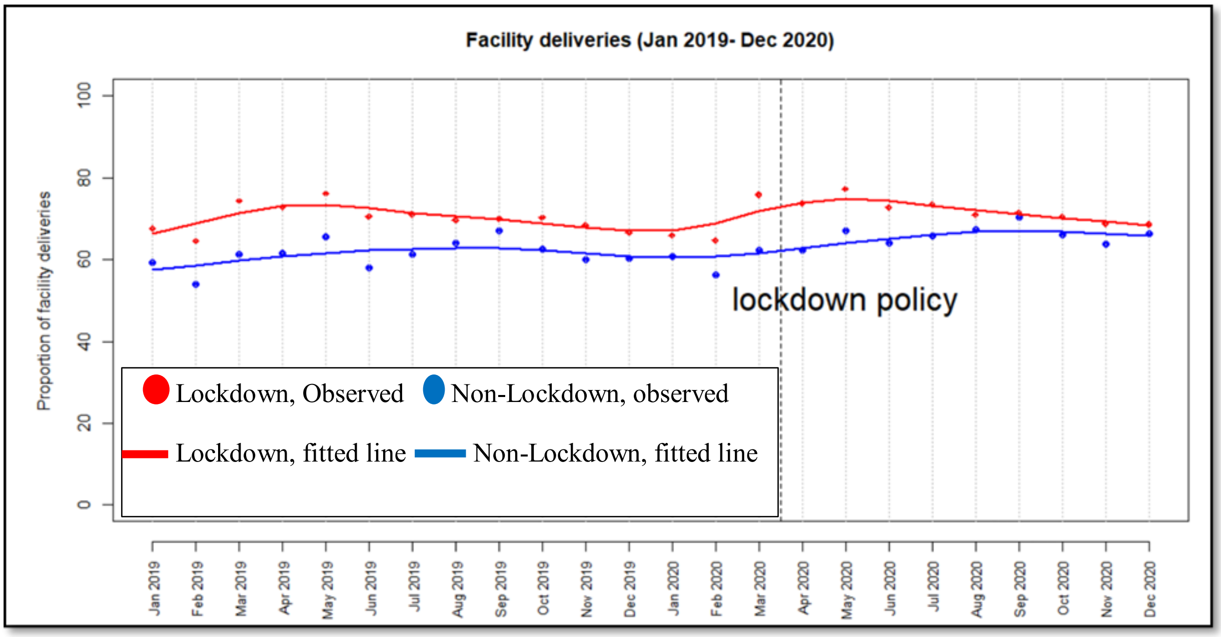

Third, we tried to adjust for health workers' strike as a dummy variable in December 2020. The rate of facility-based delivery reduced the effect size of the lockdown policy although the p-values are not significant.

Appendix 9 Model estimates-adjusting for health worker strikes only

**Pre-policy period**		
Baseline level	**Estimates [95% CI] (100%)**	**P- value**
Difference between lockdown and non-lockdown counties (β_2)	13.77 [12.31, -15.24]	P < 0.00***
Non-lockdown counties (β_0)	60.88 [59.83, 61.92]	P < 0.00***
**Pre-existing trend**		
Difference between lockdown and non-lockdown counties (β_3)	-0.26 [-0.44, -0.08]	P < 0.01**
Non-lockdown counties (β_1)	0.09 [-0.04, 0.22]	P < 0.16
**Post-policy**		
**Effect of lockdown policy (Difference between lockdown and non-lockdown)**		
Level change (β_4)	1.37 [-3.04, 5.78]	P < 0.55
Trend change (β_5)	-0.48[-1.08, 0.11]	P < 0.12
**Lockdown period (non-lockdown counties)**		
Level change (β_6)	0.84 [-2.49, 4.18]	P < 0.62
Trend change (β_7)	0.37 [-0.12, 0.85]	P < 0.15
Health worker strike	2.81 [-2.65, 8.26]	P < 0.32

Significance level at, 0.01*, and 0.05**, 0.001 ***respectively

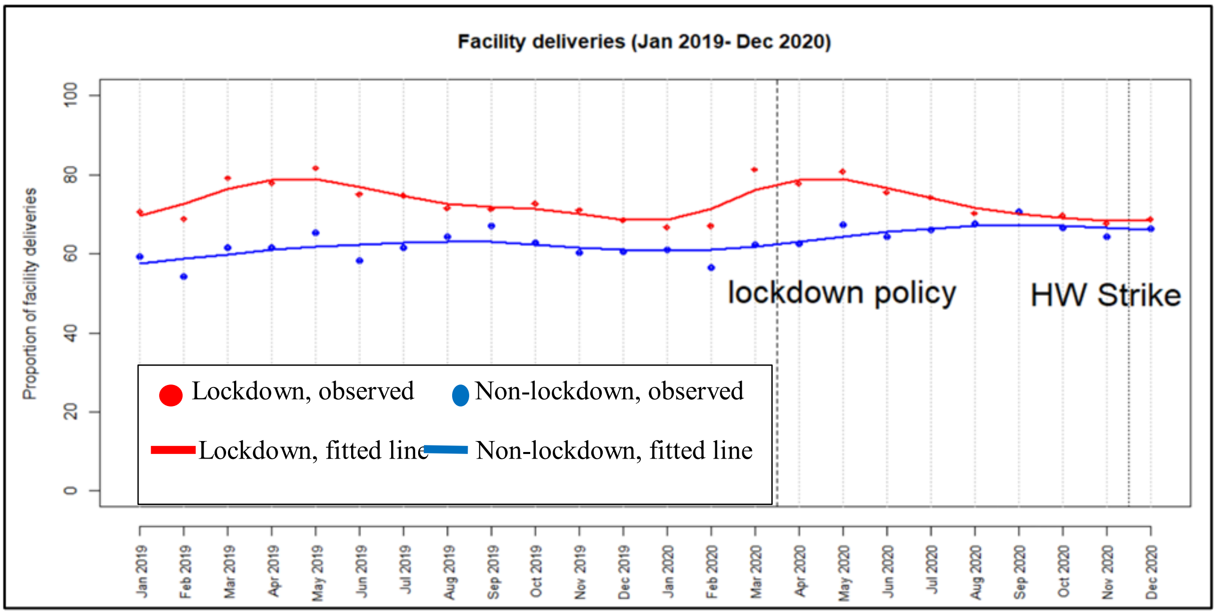

Appendix 10 Model estimates with the quadratic model term

**Pre-policy**		
**Baseline level**	**Estimates [95% CI] (100%)**	**P- value**
Before lockdown policy		
Difference between lockdown and non-lockdown counties (β_2)	14.12[12.63, 15.61]	P < 0.00***
Non-lockdown counties (β_0)	60.60[59.55, 61.66]	P < 0.00***
**Pre-existing trend**		
Difference between lockdown and non-lockdown counties (β_3)	-0.31[-0.49, -0.12]	P < 0.00**
Non-lockdown counties (β_1)	0.14[0.01, 0.27]	P < 0.05*
**Post-Policy**		
**Effect of lockdown policy (Difference between lockdown and non-lockdown counties)**		
Level change (β_4)	1.68[-7.17, 10.53]	P < 0.71
Trend change (β_5)	0.20[-3.53, 3.93]	P < 0.92
Int_trendsq	-0.10[-0.48, 0.27]	P < 0.59
**Lockdown period (non-lockdown counties)**		
Level change (β_6)	-2.92[-9.18, 3.34]	P < 0.37
Trend change (β_7)	1.63[-1.01, 4.26]	P < 0.23
Trendsq	-0.092 [-0.36, 0.17]	P < 0.499

Significance level at, 0.01*, and 0.05**, 0.001 ***respectively

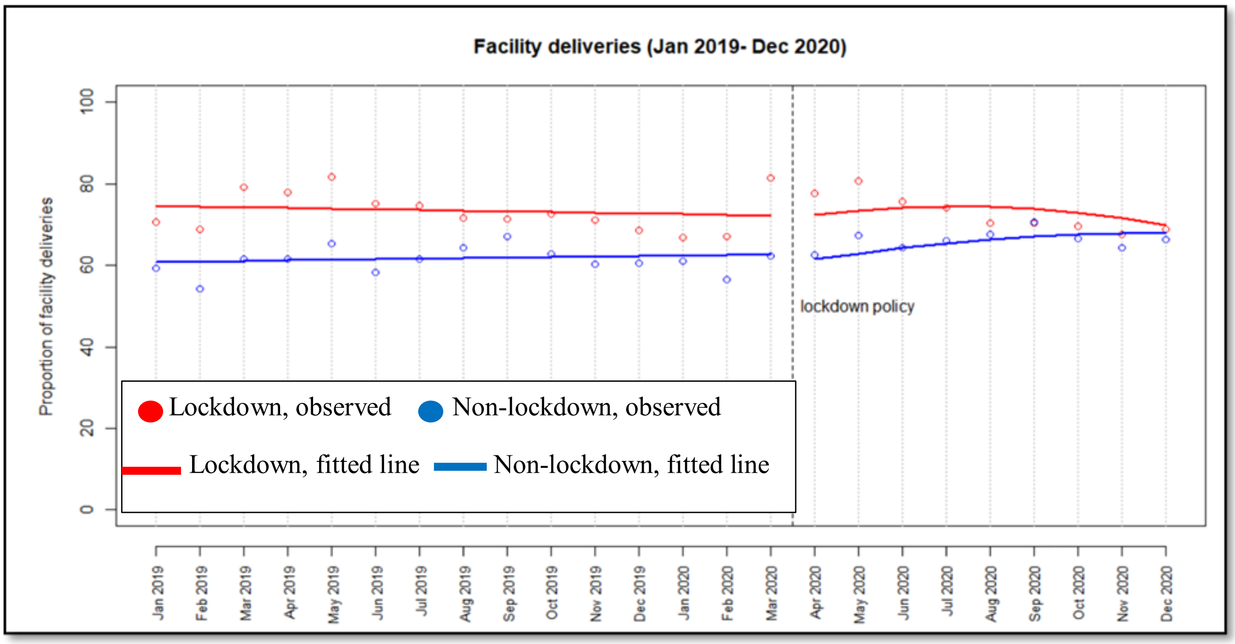
Facility deliveries (Jan 2019- Dec 2020)
Proportion of facility deliveries
0
20
40
60
80
100
lockdown policy
Lockdown, observed
Non-lockdown, observed
Lockdown, fitted line
Non-lockdown, fitted line
Jan 2019
Feb 2019
Mar 2019
Apr 2019
May 2019
Jun 2019
Jul 2019
Aug 2019
Sep 2019
Oct 2019
Nov 2019
Dec 2019
Jan 2020
Feb 2020
Mar 2020
Apr 2020
May 2020
Jun 2020
Jul 2020
Aug 2020
Sep 2020
Oct 2020
Nov 2020
Dec 2020
